# Construction of nomogram model based on contrast-enhanced ultrasound parameters to predict the degree of pathological differentiation of hepatocellular carcinoma

**DOI:** 10.3389/fonc.2025.1519703

**Published:** 2025-01-27

**Authors:** Shu-Min Lian, Hong-Jing Cheng, Hong-Jing Li, Hui Wang

**Affiliations:** ^1^ Department of Ultrasound, China-Japan Union Hospital of Jilin University, Changchun, Jilin, China; ^2^ Department of Pathology, China-Japan Union Hospital of Jilin University, Changchun, Jilin, China

**Keywords:** nomogram, contrast-enhanced ultrasound, primary hepatocellular carcinoma, Edmondson-Steiner grade, VueBox^®^ external perfusion software

## Abstract

**Objective:**

To predict the degree of pathological differentiation of hepatocellular carcinoma (HCC) by quantitative analysis the correlation between the perfusion parameters of contrast-enhanced ultrasound (CEUS) and the pathological grades of HCC using VueBox^®^ software.

**Methods:**

We enrolled 189 patients who underwent CEUS and liver biopsy at our hospital from July 2019 to September 2024 and were pathologically confirmed with primary HCC. The Edmondson-Steiner pathological classification system was used as the gold standard for dividing the patients into the low-grade and high-grade groups. The patients were randomly divided into training set and testing set in a ratio of 7:3, in which the parameters of the training set were analyzed by univariate analysis and then stepwise regression to construct the prediction model, and the diagnostic efficacy of the validation model was evaluated by discrimination, calibration, and clinical applicability.

**Results:**

A total of 189 patients with primary hepatocellular carcinoma were enrolled, including 118 patients in the low-grade group and 71 patients in the high-grade group; they were randomly divided into training set of 128 patients and testing set of 61 patients. The prediction model was constructed by logistic regression in the training set, and the final model included three variables: mTTI, FT, and maximum diameter of a single lesion, resulting in the equation was $Y=-2.360+1.674X_{1}+1.019X_{2}+0.753X_{3\left(2\right)}+1.570X_{3\left(3\right)}$ .The area under the ROC curve (AUC) of the training set was 0.831, with a sensitivity of 82.0% and a specificity of 79.5%; the area under the ROC curve (AUC) of the testing set was 0.811, with a sensitivity of 81.0% and a specificity of 70.0%.

**Conclusion:**

The regression model constructed by combining multiple parameters can effectively improve the diagnostic performance of CEUS in predicting the pathological differentiation grade of HCC, thus providing a clinical basis and empirical support for the use of CEUS as a diagnostic imaging method for this disease.

## 1 Introduction

HCC is characterized by high morbidity and high mortality. Although a variety of comprehensive treatments can be used to treat HCC effectively, the 5-year patient survival rate remains very low (1). Therefore, choosing a suitable treatment for HCC patients depending on the tumor stage can maximize treatment efficacy and obtain a better prognosis. The pathological differentiation of HCC plays an important role in the clinical staging of liver cancer and is helpful for the clinical development of treatment strategies. Surgical resection, liver transplantation, and tumor ablation are usually selected for HCC with good pathological differentiation, while radiotherapy combined with systemic anti-tumor therapy is considered for HCC with poor pathological differentiation in order to have the possibility of radical resection in the future (2). At the same time, it has been shown that HCC with better pathological differentiation also has a better prognosis, while HCC patients with worse pathological differentiation have a worse prognosis (3). Traditional pathological differentiation grading and classification depend on preoperative biopsy and postoperative pathological diagnosis, which are invasive and limited by their inherent sampling biases; however, the noninvasive, preoperative evaluation of the pathological classification of HCC remains challenging. Ultrasound examination is the preferred imaging examination method for screening patients for HCCs and has the advantages of convenience, real-time, noninvasive, and radiation-free. In recent years, the new technology of CEUS can dynamically detect the contrast agent in real time and the tissue microcirculation through continuous imaging of mechanical index, providing strong evidence for qualitatively determining tumor characteristics and information on the differentiation of tumor tissues (4). The real-time CEUS manifestations of HCC is influenced by the pathological grading of the tumor, and its perfusion parameters have a high correlation with the pathological grading of the tumor, which can be used to make a preliminary judgment on the pathological differentiation of HCC (5). Preoperative predicting the pathological differentiation grade of HCC by CEUS can provide more reference for clinicians to develop a reasonable treatment plan and has certain clinical significance.

As an important part of CEUS, the safety of contrast agents has been the focus of clinical attention. The contrast agent used in this study is SonoVue^®^ (Bracco, Italy), and it can be removed from the body via the pulmonary circulation and is safe, it can be used even in children, patients with impaired renal function and patients with hyperthyroidism (6–10). However, analyses of intralesional ultrasound contrast agent perfusion are still determined by the sonographer, making the results relatively subjective. This study used the independent external perfusion analysis software VueBox^®^ (Bracco, Italy), an offline, universal perfusion analysis software for CEUS. Unlike the time-intensity curve (TIC) analysis performed by the integrated perfusion software used on high-end ultrasound machines, the use of SonoVue^®^ as a contrast agent and the application of the VueBox^®^ software for the analysis after properly calibrating the machine and probe can correct for movement artifacts and parametrically display pseudo-colors and therefore provide a more objective view of the various parameters of the vascular distribution of the tumor and quantify tumor perfusion, effectively eliminating the effects of human subjectivity (11–15). VueBox ^®^ perfusion analysis software has relatively more quantitative indicators, which also effectively solves the problem of limited quantitative indicators of ultrasound machine with perfusion software, making the research results more scientific. In this study, by investigating the correlation between quantitative CEUS parameters and the pathological classification of HCC, we constructed an ultrasound prediction model to noninvasively predict the pathological differentiation grade of HCC preoperatively and provide convenience for clinical diagnosis and treatment of HCC.

## 2 Materials and methods

### 2.1 Research subjects

This study was a single-center retrospective study and data from all participating patients were anonymous. And this study was approved by the Research Ethics Committee and the requirement for informed consent was waived.

A total of 189 patients (189 lesions) who underwent routine ultrasound and CEUS examinations and underwent liver biopsy and were pathologically confirmed to have primary hepatocellular carcinoma after surgery in our hospital from July 2019 to September 2024 were retrospectively analyzed. We have formulated the corresponding inclusion and exclusion criteria, where the inclusion criteria were as follows: 1) pathologically confirmed HCC after percutaneous liver biopsy (PLB); 2) complete CEUS images and DICOM-format angiographic images; And 3) complete basic clinical information, such as sex, age, and Edmondson-Steiner pathological classification. The exclusion criteria were as follows: 1) local or systemic liver treatments, such as transcatheter arterial chemoembolization (TACE), targeted immunotherapy, partial hepatectomy, chemoradiotherapy, and local ablation, prior to liver CEUS; 2) incomplete DCE-US images; 3) images that could not be analyzed by angiography analysis software due to the presence of respiration-related movement artifacts.

After determining the study population, patients were divided into low-grade group (118 patients) and high-grade group (71 patients) using Edmondson-Steiner pathological grade as the gold standard. Edmondson-Steiner pathological grades are mainly divided by cell morphology, arrangement pattern, nucleoplasmic ratio, and mitotic figures. I: The morphology of cancer cells was similar to that of normal hepatocytes, arranged in cords, with eosinophilic cytoplasm, regular nuclear size, and very few mitotic figures; II: Mild morphological changes of cancer cells, cord-like or nested arrangement, significantly increased nucleoplasmic ratio, increased mitotic figures; III: Cancer cells were significantly deformed and arranged in nests, the nucleus-to-cytoplasm ratio continued to increase, the nuclei varied in size, the staining was also irregular, mitotic figures were more common, and giant cancer cells were sometimes observed; IV: Cancer cells showed obvious atypia, showing spindle cell multinucleated giant cells, scant cytoplasm and deep nuclear staining, mitotic figures were more common, and cells were disorganized (16). The low-grade group corresponds to grades I-II and the high-grade group corresponds to grades III-IV (17, 18).

### 2.2 Instruments and methods

Ultrasound examinations of all patients were performed independently by a ultrasound physician with more than 10 years of working experience, using a convex array probe 3.5-5.0 MHz ultrasound diagnostic apparatus (mindry Resona 7, mindry Resona 8, mindry Resona R9 Exp).

First, a conventional color Doppler ultrasound examination was performed. The operator provided the examinee with breathing instructions to determine and record the location, morphology, structure, size (maximum diameter), echo signal, and blood flow of the tumor.

**Figure 1:**
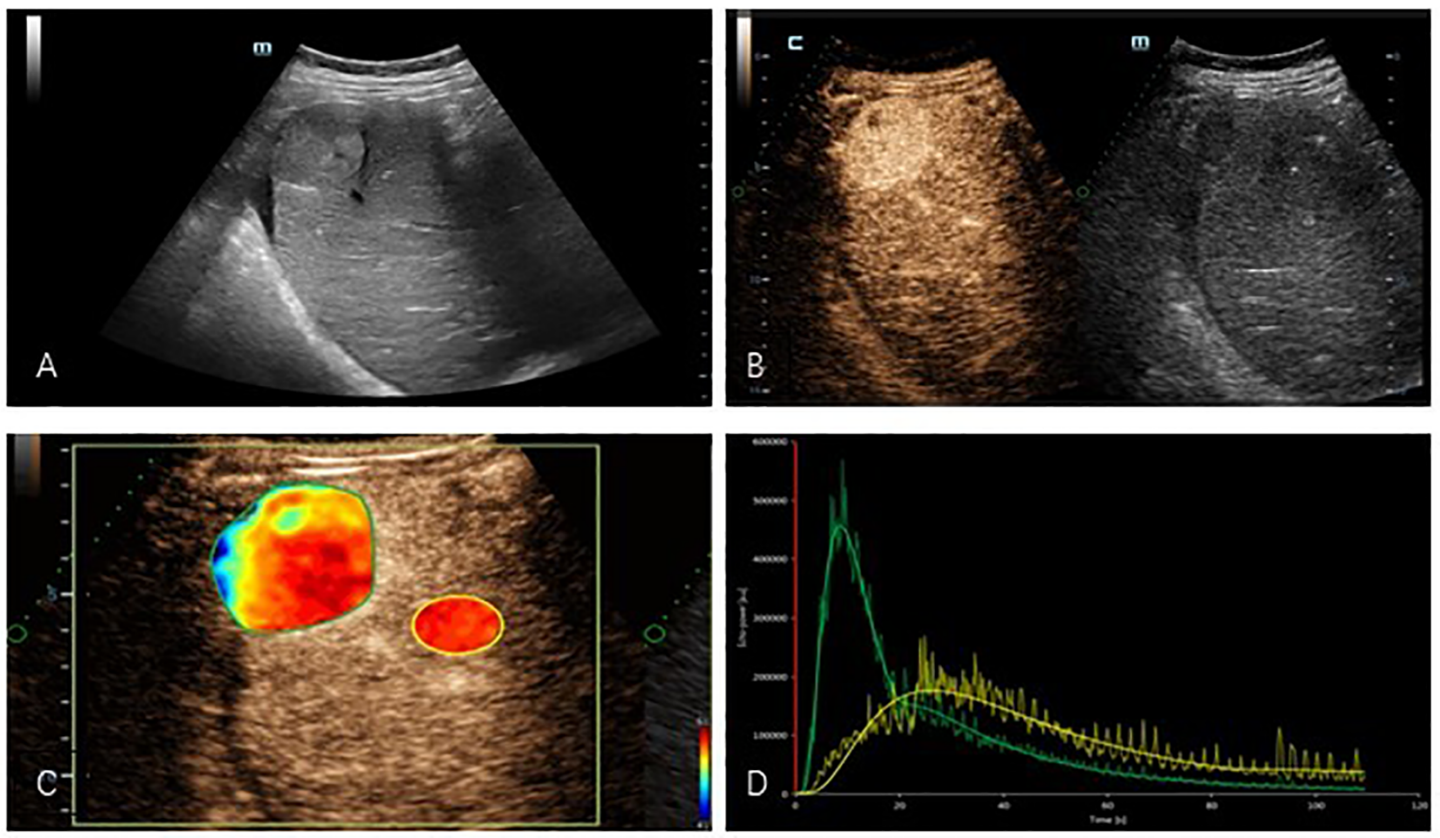
**(A)** Two-dimensional ultrasound showed a hyperechogenicity in the right lobe of the liver with well-defined borders and less homogeneous internal echoes. **(B)** Contrast-enhanced ultrasound images showed hyperenhancement in the arterial phase of the tumor. **(C)** Regions of interest were delineated in quantitative analysis images of masses. **(D)** TIC: Compared with normal liver tissue, the tumor presents a typical appearance as “fast wash-in and fast wash-out”. (yellow: normal liver tissue, green: mass).

Subsequently, the CEUS examination was performed. The ultrasound system was switched to angio-mode, and 2.4 mL of a SonoVue^®^ microbubble suspension was injected through the median vein of the elbow, followed by washout with 5-10 ml of 0.9% normal saline. The perfusion of contrast agent within the lesion was observed for 5 minutes, and the complete image sequence was saved. The recording included three phases: the arterial phase (10-30 s after the start of injection), portal venous phase (30-120 s after the start of injection) and delayed phase (>120 s). The images were exported in DICOM format. The area with the most significant macroscopic enhancement of the lesion was selected as the region of interest for delineation, and attention was paid to avoid areas of intratumoral hemorrhage, cystic degeneration, and necrosis. Subjected to DCE-US analysis with VueBox^®^ software (Bracco). The following parameters were obtained from the TIC: average contrast signal intensity (MeanLin), peak enhancement (PE), wash-in area under the curve (WiAUC), rise time (RT), mean transit time (mTTl), time to peak (TTP), wash-in rate (WiR), wash-in perfusion index (WiPI), wash-out area under the curve (WoAUC), wash-in and wash-out area under the curve (WiWoAUC), fall time (FT), and wash-out rate (WoR) (**Figure 1**). The specific significance of each indicator is as follows. MeanLin: Mean signal intensity of perfusion in the region of interest. PE: Signal intensity at the time of highest perfusion in the region of interest, reflecting the blood volume in the region of interest. WiAUC: Area under the ascending limb of the curve, reflecting inflow perfusion and blood velocity in the region of interest. RT: Time between arrival of contrast agent and peak. mTTl: Time from contrast agent wash in to 50% wash out. TTP: Time elapsed from perfusion to peak intensity. WiR: It is related to the maximum blood perfusion flow and perfusion time, reflecting the speed of blood perfusion in the region of interest. WiPI: Area under the inflow phase curve divided by rise time. WoAUC: Area under the descending branch of the curve, associated with outflow phase flow clearance. WiWoAUC: Area under the ascending and descending branches of the curve, associated with perfusion and clearance. FT: Time between peak and contrast agent falling to basal intensity. WoR: Reflects the speed of blood flow clearance in the region of interest.

### 2.3 Statistical methods

The obtained data were analyzed using IBM SPSS Statistics 27.0 software. Normally distributed data are expressed as the $\;\bar{x}\pm s$ , and the differences between groups were assessed using the t test. Data that did not conform to a normal distribution are expressed as the M (Q1, Q3), and the differences between groups were assessed using the Mann-Whitney U test.

**Figure 2:**
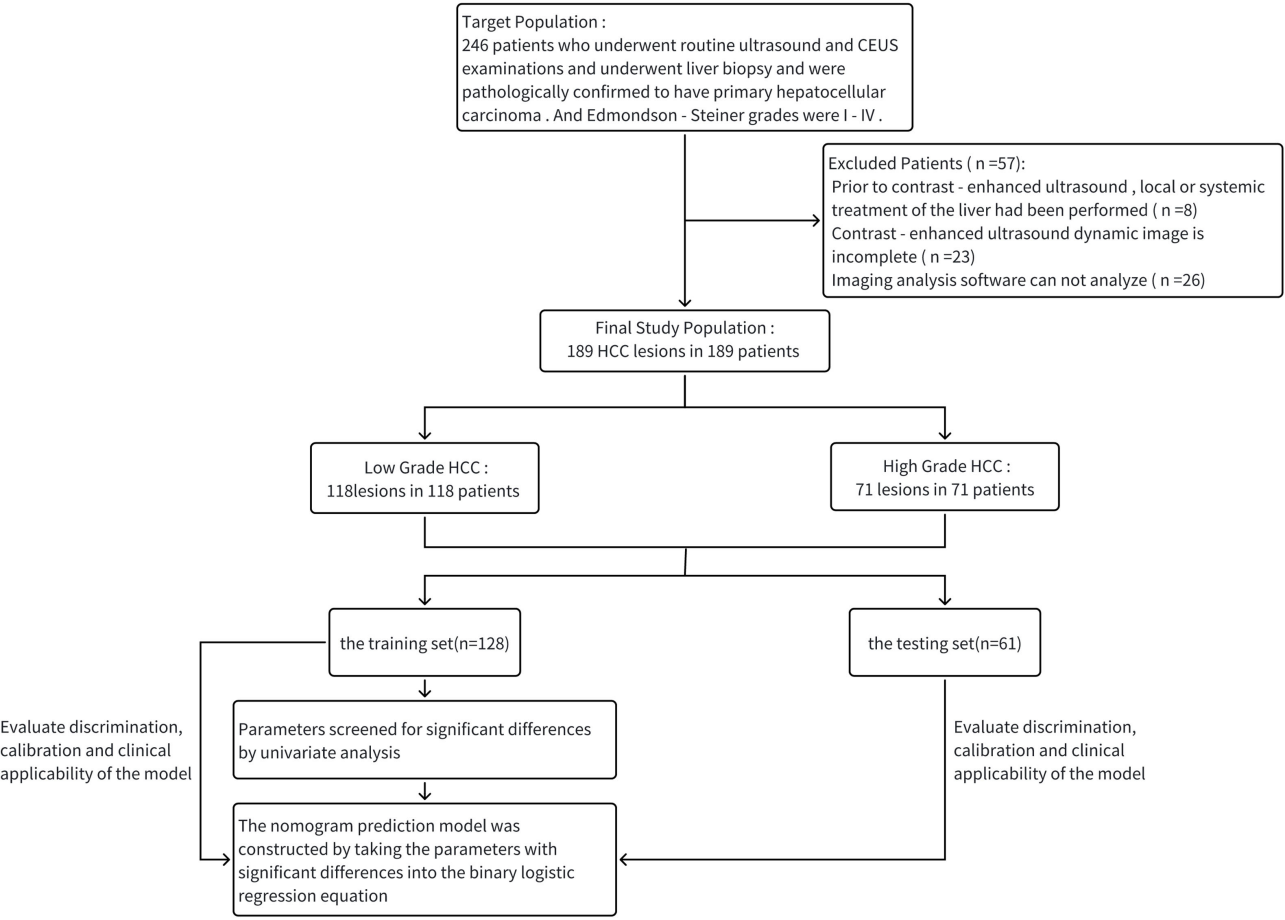
The flow chart of the whole experiment.

Enumeration data were expressed as frequency and percentage, χ^2^ test was used to compare differences between groups. Differences were considered to be statistically significant if the p <0.05. With software R4.3.3, the data were randomly divided into training set (128 cases) and testing set (61 cases) in a 7:3 ratio by setting a random seed number. Then the parameters with significant differences in univariate analysis in the training set were brought into the binary logistic regression equation to construct the prediction model, and the nomogram of the prediction model was drawn. ROC and Hosmer-Lemeshow test were used to evaluate the discrimination and calibration of the constructed model in the training set and testing set, and the benefit and applicability of the model when applied in clinical practice were assessed by the decision curve (**Figure 2**).

## 3 Results

### 3.1 Patient information and comparison between training and testing set

**Table 1** describes the basic information of patients in the entire cohort. **Table 2** shows the comparative analysis of the data between the training set and the testing set, including the training set (128 cases in total, 78 cases in the low-grade group, and 50 cases in the high-grade group) and the testing set (61 cases in total, 40 cases in the low-grade group, and 21 cases in the high-grade group). The statistical analysis showed that there were no significant differences in the parameters between the two groups (P > 0.05), which could be used for the construction and validation of the model.

**Table 1:** Basic data of patients with hepatocellular carcinoma.

Characteristic	Total n=189	Low-grade hepatocellular carcinoma (Edmondson-Steiner grades I and II) n=118	High-grade hepatocellular carcinoma (Edmondson-Steiner grades III and IV) n=71
Age/(year)	60.00 (51.50,68.00)	62.00 (51.75,69.00)	57.00 (49.00,64.00)
Gender/n (%)			
Male	135 (71.4)	82 (69.5)	53 (74.6)
Female	54 (28.6)	36 (30.5)	18 (25.4)
MeanLin/dB	46.67 (39.24,51.06)	47.17 (40.34,51.13)	44.97 (38.35,48.94)
PE/dB	49.74 ± 8.51	49.86 ± 8.69	49.53 ± 8.26
WiAUC/dB	58.01 ± 8.52	58.67 ± 8.48	56.92 ± 8.54
RT/s	8.28 (6.75,13.31)	8.62 (7.09,14.35)	7.75 (6.33,10.24)
mTTI/s	52.97 (35.21,84.00)	57.78 (49.50,91.02)	40.29 (30.17,75.76)
TTP/s	13.14 (10.10,17.04)	13.70 (10.17,17.71)	12.16 (9.96,16.58)
WiR/dB	43.72 (35.25,48.47)	43.50 (36.15,48.13)	43.72 (33.52,48.79)
WiPI/dB	49.50 (42.25,53.25)	49.78 (42.68,54.01)	47.81 (41.88,52.67)
WoAUC/dB	61.85 ± 8.69	62.57 ± 8.67	60.66 ± 8.64
WiWoAUC/dB	63.32 ± 8.60	63.97 ± 8.49	62.23 ± 8.74
FT/s	19.08 (13.78,33.18)	22.73 (16.49,37.85)	15.52 (10.78,20.93)
WoR/dB	37.24 ± 9.88	36.76 ± 9.80	38.03 ± 10.01
diameter/cm	3.60 (2.00,5.30)	2.70 (1.80,4.30)	4.40 (3.10,7.50)

**Table 2:** Comparison analysis of parameters between training set and testing set.

Parameter	Training set (n=128)	Testing set (n=61)	P
Age/(year)	59.30 ± 10.89	59.89 ± 10.37	0.700
Gender/n (%)			0.623
Male	90 (70.3)	45 (73.8)	
Female	38 (29.7)	16 (26.2)	
MeanLin/dB	44.77 ± 8.49	47.18 ± 7.61	0.238
PE/dB	49.68 ± 8.96	49.85 ± 7.55	0.242
WiAUC/dB	57.30 ± 8.93	59.50 ± 7.45	0.229
RT/s	7.99 (6.63,12.59)	8.68 (6.92,14.07)	0.349
mTTI/s	51.73 (33.75,88.20)	55.15 (37.41,78.18)	0.976
TTP/s	12.94 (9.98,16.97)	13.76 (10.47,17.10)	0.268
WiR/dB	42.01 ± 9.47	41.74 ± 8.66	0.563
WiPI/dB	47.79 ± 8.92	49.26 ± 7.64	0.139
WoAUC/dB	60.99 ± 9.02	63.66 ± 7.69	0.287
WiWoAUC/dB	62.53 ± 8.90	64.96 ± 7.74	0.362
FT/s	18.91 (12.71,33.84)	19.98 (14.22,32.11)	0.698
WoR/dB	36.92 ± 10.15	37.91 ± 9.32	0.329
diameter/cm	3.70 (2.10, 5.30)	3.10 (2.00,4.80)	0.285

### 3.2 Single factor analysis of training set parameters

The results of univariate analysis in the training set showed that mTTI, FT, and maximum diameter of single lesions (hereafter referred to as diameter) were significantly different among patients with different differentiation degrees; the remaining variables (age, gender, MeanLin, PE, WiAUC, RT, TTP, WiR, WiPI, WoAUC, WiWoAUC, and WoR) were not (**Table 3**).

**Table 3:** Single factor analysis of each parameter in the training set.

Indicators	Low-grade Group (n=78)	High-grade Group (n=50)	P
Age/(year)	61.12 ± 11.25	56.46 ± 9.76	0.092
Gender/n (%)			0.738
Male	54 (69.2)	36 (72.0)	
Female	24 (30.8)	14 (28.0)	
MeanLin/dB	45.04 ± 8.75	44.34 ± 8.14	0.651
PE/dB	49.71 ± 9.14	49.64 ± 8.76	0.768
WiAUC/dB	57.66 ± 9.12	56.74 ± 8.68	0.647
RT/s	8.62 (6.64,14.17)	7.59 (6.27,10.19)	0.057
mTTl/s	59.00 (49.59,94.73)	40.06 (29.92,76.01)	<0.001
TTP/s	13.76 (10.02,18.53)	11.70 (9.82,15.85)	0.282
WiR/dB	41.85 ± 9.49	42.26 ± 9.52	0.867
WiPI/dB	47.85 ± 9.10	47.71 ± 8.73	0.782
WoAUC/dB	61.63 ± 9.25	59.99 ± 8.65	0.582
WiWoAUC/dB	63.06 ± 9.08	61.71 ± 8.65	0.648
FT/s	21.96 (17.37,38.26)	15.62 (10.98,21.99)	<0.001
WoR/dB	36.39 ± 10.20	37.74 ± 10.12	0.784
diameter/cm	2.80 (1.90,4.70)	4.40 (3.10,7.50)	<0.001

### 3.3 Binary logistic regression analysis

To avoid the loss of accuracy in establishing regression models due to possible high correlation between independent variables, collinearity analysis was performed using variance inflation factor (VIF) for three variables: mTTI, FT, and diameter. The results showed that the VIF values were less than 10, indicating that there was no collinearity problem between the independent variables to be included in the regression analysis.

Data in the training set used pathological results as dependent variable (0, Edmondson-Steiner grade I-II; 1, Edmondson-Steiner grade III-IV), mTTI, FT, and diameter, which were significantly different between the groups in the univariable analysis (**Table 3**), were considered independent variables. The diagnostic cut-off values of mTTI and FT were determined using the Youden index (48.89 s and 17.64 s, respectively) and used to assign the patients a criteria for subgroup groups of 0 if their value was lower than the cut-off value and of 1 otherwise; according to the Chinese clinical staging criteria for liver cancer (2), the diameters were divided into three groups of 0, 1, and 2 if the value was ≤3 cm, 3-5 cm, and > 5 cm, respectively. Finally, these groups were used to perform binary logistic regression analysis, and the following regression equation was obtained: $Y=-2.360+1.674X_{1}+1.019X_{2}+0.753X_{3\left(2\right)}+1.570X_{3\left(3\right)}$ . Analysis of the results of the equation (**Table 4**) indicated that the likelihood of an HCC lesion with an mTTI <48.89 s being in the high-grade group was 5.335 times that of one with an mTTI ≥ 48.89 s; the likelihood of an HCC lesion with an FT <17.64 s being in the high-grade group was 2.770 times that of one with an FT ≥ 17.64 s; finally, among the lesion diameter groups, a significant difference was only identified between those with a diameter ≤3 cm and those with a diameter >5 cm, and the likelihood that a tumor with a diameter >5 cm would be in the high-grade group was 4.806 times greater than that of a tumor with a diameter ≤ 3 cm.

**Table 4:** Results of multi-parameter binary logistic regression analysis.

Factor	β	SE	df	p	OR	95%CI	
						Lower limit	Upper limit
*X*_1_ mTTI Group	1.674	0.510	1	0.001	5.335	1.962	14.507
*X*_2_ FT Group	1.019	0.502	1	0.042	2.770	1.036	7.409
*X*_3_ Diameter			2	0.023			
Diameter ≤3cm					1.000		
Diameter 3-5cm	0.753	0.538	1	0.161	2.124	0.740	6.092
Diameter >5cm	1.570	0.570	1	0.006	4.806	1.572	14.698
Constant	-2.360	0.459	1	<0.001	0.094		

### 3.4 Construction of the nomogram prediction model

**Figure 3:**
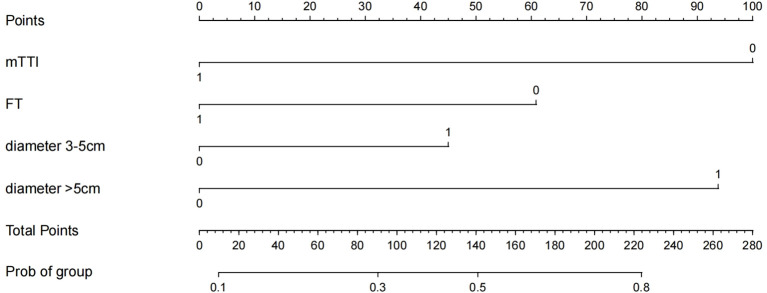
Nomogram predictive model.

Based on the results of logistic regression analysis, the software R4.3.3 was used to construct a nomogram prediction model, as shown in **Figure 3**. The nomogram score for HCC lesions with an mTTI <48.89 s was 100 points greater than that of lesions with an mTTI ≥48.89 s; HCC lesions with an FT<17.64 s scored 60 points higher than lesions with an FT ≥17.64 s; and lesions with a diameter >5 cm scored 94 points higher than those with a diameter ≤ 3 cm. That is, the higher the score, the higher the risk that the tumor may be of a high grade.

### 3.5 Evaluation of the diagnostic performance of the nomogram model

#### 3.5.1 Discrimination evaluation

**Figure 4:**
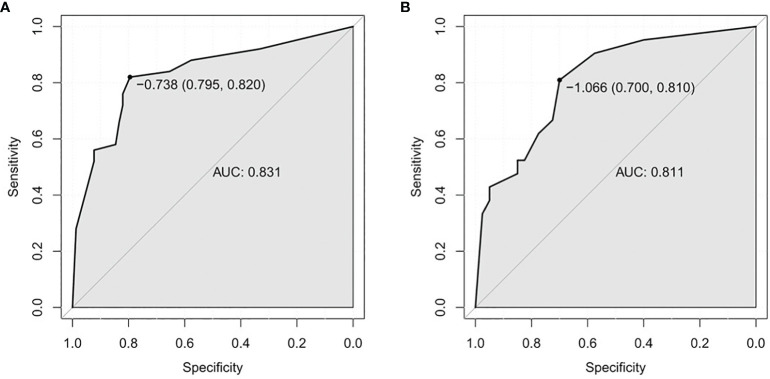
ROC curves. **(A)** Training set. **(B)** Testing test. ROC, receiver operating characteristic; AUC, area under the ROC curve.

In the univariate analysis, mTTI yielded an AUC of 0.754, a sensitivity of 70.0%, and a specificity of 80.8%; for the FT, the AUC was 0.728, the sensitivity was 70.0%, and the specificity was 75.6%; for the diameter, the AUC was 0.692, the sensitivity was 80.0%, and the specificity was 51.3%. While the AUC of this model in the training set was 0.831, with a sensitivity of 82.0% and a specificity of 79.5%, and the AUC of this model in the testing set was 0.811, with a sensitivity of 81.0% and a specificity of 70.0%, indicating that the accuracy of this model in predicting the pathological differentiation of HCC was high and significantly better than each factor (**Table 5**; **Figure 4**).

**Table 5:** Comparison of diagnostic efficacy between univariate and predictive models in the training set.

Parameter	AUC	Sensitivity	Specificity
mTTI	0.754	70.0%	80.8%
FT	0.728	70.0%	75.6%
Diameter	0.692	80.0%	51.3%
Logistic regression model	0.831	82.0%	79.5%

#### 3.5.2 Calibration evaluation

**Figure 5:**
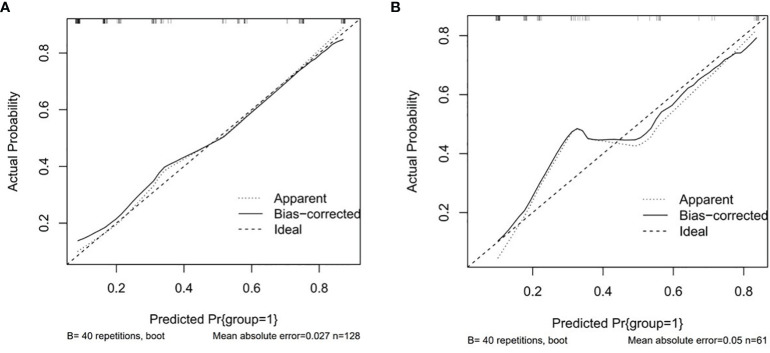
Calibration curves. **(A)** Training set. **(B)** Testing test.

The P values of Hosmer-Lemeshow test were 0.104 and 0.848 in the training set and testing set, respectively, p > 0.05, indicating that the model has a good fit. Drawing the calibration curve to visualize the goodness of fit results of the Hosmer-Lemeshow test showed that the slope of the calibration curve was nearly 1 in both the training set and the testing set, indicating that the consistency between the predicted pathological differentiation grade by the model and the actual grade was good (**Figure 5**).

#### 3.5.3 Clinical applicability evaluation

**Figure 6:**
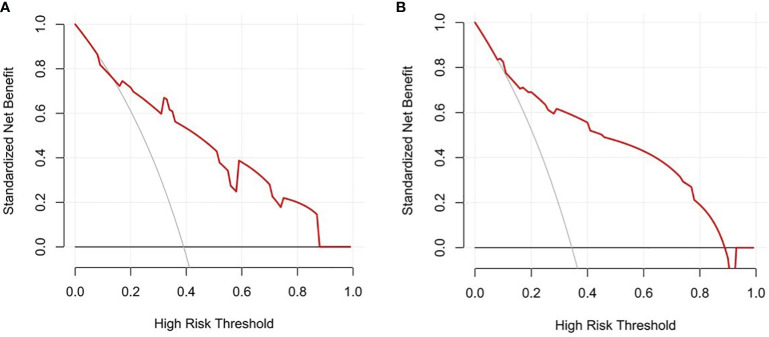
Decision curves. **(A)** Training set. **(B)** Testing test.

Decision curves result for the training and testing sets showed that the model yielded greater net benefits than potential treat-all and treat-none strategies at threshold probabilities ranging from 0.1 to 0.9 (**Figure 6**).

## 4 Discussion

CEUS plays an increasingly important role in the diagnosis and treatment of HCC. On CEUS, HCC often demonstrates rapid wash-in in the arterial phase and rapid wash-out in the portal venous phase and delayed phase, also known as “fast wash-in and fast wash-out”, which is more common in HCCs with moderate and poor differentiation, while some hyperdifferentiated HCCs show “fast wash-in and slow wash-out or not wash-out” on CEUS (19). It is possible that due to moderately and poorly differentiated HCC is mainly supplied by the hepatic artery, the contrast agent rapidly enters the tumor in the arterial phase, and the contrast agent is basically washed out from the tumor in the portal venous phase, while the non-tumor liver parenchyma begins to enhance at this time, so the tumor appears hypoechoic relative to the surrounding liver.

Rhee H. et al. (20) concluded that early HCC usually does not show or only a small proportion shows hyperenhancement in the arterial phase. This is because in the early stages of HCC, invasive growth of tumor cells destroys preexisting vascular structures, accompanied by incomplete neovascularization and sinusoidal capillary formation. So arterial phase hyperenhancement is usually not used as a basis for the diagnosis of early HCC.

Contrast-enhanced ultrasonography is no longer limited to exploring the correlation between enhancement pattern and histopathology, and it has been shown that the quantitative parameters of CEUS also have a good correlation with histopathological grade of HCC. Xu J. F. et al. (21) concluded that the TTP, contrast-enhanced time, wash-out time, enhancement slope, and clearance slope of HCC were significantly correlated with tumor stage. Xuan Z. et al. (22) showed that significant differences were present in the RT, TTP, enhancement rate and mean diameter of HCC tumors with different differentiation degrees on CEUS, consistent with the findings of this study, in which the FT and tumor diameter were significantly different among HCC patients with different degrees of differentiation. Jang H. J. et al. (23) believed that as tumors develop from well-differentiated to moderately differentiated to poorly differentiated, the contrast agent elutes earlier. Salvatore V. et al. (24) also concluded that the wash-out time was negatively correlated with the degree of differentiation of the tumor. In this study, the Youden index was used to determine the diagnostic cutoff values of the abovementioned parameters. The analysis showed that HCC lesions with an FT less than the cutoff value of 17.64 s were 2.770 times more likely to be poorly differentiated than those with a larger FT, i.e. the smaller the FT, the more poorly differentiated the tumor is likely to be, consistent with the findings of previous scholars. Although tumor size (diameter) is a clinical staging factor for HCC, in this study, it was not linearly related to the pathological differentiation degree. Instead, significant differences were found in the degree of differentiation of HCC only between tumors ≤ 3 cm and >5 cm in diameter, with the latter considered more likely to be poorly differentiated. HCC > 5 cm is considered to be more prone to tumor invasion as well as distant metastasis in clinical (3). MTTI is a unique quantitative parameter in VueBox^®^ software and has not been mentioned in previous correlation studies. Our analysis showed that the smaller the value of mTTI, the worse the tumor differentiation. And as a single factor, mTTI had better diagnostic efficacy (AUC = 0.754), significantly better than FT (AUC = 0.728) and tumor diameter (AUC = 0.692). FT and mTTI are independent predictors for predicting the pathological grade of HCC and play an important role in distinguishing HCC levels with different degrees of differentiation. This may be because during the development of HCC, it is accompanied by changes in vascular supply, drainage, and microvascular structure. In the early stages of HCC, stromal invasion leads to portal vein destruction, vascular supply is reduced, and unpaired arteries begin to develop. To moderately and poorly differentiated HCC, the portal triad almost completely disappears, and the number of unpaired arteries increases significantly, which may be accompanied by the formation of intratumoral arteriovenous fistulas. This series of tumor perfusion changes allows the contrast agent to remain in poorly differentiated tumors for a shorter period of time than well-differentiated ones.

The AUC values of the models constructed in this study were 0.831 (training set) and 0.811 (testing set), indicating good discriminant ability. However, the sensitivity and specificity of the two groups showed some differences (82.0%/79.5% in the training set and 81.0%/70.0% in the testing set), and the reason for the relatively low specificity in the testing set may be related to the different proportion of patients in the training set and the testing set in the high-grade group. This study included a small sample size and was conducted with the data from only a single center study. In the future, we hope to increase the sample size and perform multicenter studies to further explore and validate prediction models for different types of liver cancers.

## 5 Conclusion

Our study confirmed that the regression model constructed by combining multiple parameters can effectively improve the diagnostic performance of CEUS in predicting the pathological differentiation grade of HCC, thus providing a clinical basis and empirical support for the use of CEUS as a diagnostic imaging method for this disease.

## References

[1] Forner A Reig M Bruix J. Hepatocellular carcinoma. Lancet. (2018) 391:1301–14. doi: 10.1016/S0140-6736(18)30010-2 29307467

[2] Zhou J Sun H Wang Z Cong W Zeng M Zhou W. Guidelines for the diagnosis and treatment of primary liver cancer (2022 edition). Liver Cancer. (2023) 12:405–44. doi: 10.1159/000530495 PMC10601883 37901768

[3] Guerrini GP Pinelli D Di Benedetto F Marini E Corno V Guizzetti M. Predictive value of nodule size and differentiation in HCC recurrence after liver transplantation. Surg Oncol. (2016) 25:419–28. doi: 10.1016/j.suronc.2015.09.003 26403621

[4] Huang Z Zhou P Li S Li K. Prediction of the Ki-67 marker index in hepatocellular carcinoma based on Dynamic Contrast-Enhanced Ultrasonography with Sonazoid. Insights Imaging. (2022) 13:199. doi: 10.1186/s13244-022-01320-6 36536262 PMC9763522

[5] Loria F Loria G Basile S Crea G Randazzo D Frosina L. Contrast-enhanced ultrasound of hepatocellular carcinoma: correlation between enhancement pattern and cellular differentiation on histopathlogy. Updates Surg. (2012) 64:247–55. doi: 10.1007/s13304-012-0179-7 23055349

[6] Uggowitzer MM Gotschuli G Reiter H Petek B. Contrast-enhanced sonography of the liver. Radiologe. (2005) 45:24–33. doi: 10.1007/s00117-004-1138-1 15565382

[7] Piscaglia F Bolondi L Italian Society for Ultrasound in M Biology Study Group on Ultrasound Contrast A. The safety of Sonovue in abdominal applications: retrospective analysis of 23188 investigations. Ultrasound Med Biol. (2006) 32:1369–75. doi: 10.1016/j.ultrasmedbio.2006.05.031 16965977

[8] Dietrich CF Nolsoe CP Barr RG Berzigotti A Burns PN Cantisani V. Guidelines and good clinical practice recommendations for contrast-enhanced ultrasound (CEUS) in the liver-update 2020 WFUMB in cooperation with EFSUMB, AFSUMB, AIUM, and FLAUS. Ultrasound Med Biol. (2020) 46:2579–604. doi: 10.1016/j.ultrasmedbio.2020.04.030 32713788

[9] Claudon M Dietrich CF Choi BI Cosgrove DO Kudo M Nolsoe CP. Guidelines and good clinical practice recommendations for contrast enhanced ultrasound (CEUS) in the liver–update 2012: a WFUMB-EFSUMB initiative in cooperation with representatives of AFSUMB, AIUM, ASUM, FLAUS and ICUS. Ultraschall Med. (2013) 34:11–29. doi: 10.1055/s-0032-1325499 23129518

[10] Gomaa AI Khan SA Leen EL Waked I Taylor-Robinson SD. Diagnosis of hepatocellular carcinoma. World J Gastroenterol. (2009) 15:1301–14. doi: 10.3748/wjg.15.1301 PMC2658831 19294759

[11] Dong Y Koch JBH Lowe AL Christen M Wang WP Jung EM. VueBox(R) for quantitative analysis of contrast-enhanced ultrasound in liver tumors1. Clin Hemorheol Microcirc. (2022) 80:473–86. doi: 10.3233/CH-211261 34897079

[12] Jung EM Weber MA Wiesinger I. Contrast-enhanced ultrasound perfusion imaging of organs. Radiologe. (2021) 61:19–28. doi: 10.1007/s00117-021-00891-7 34378067 PMC8354100

[13] Platz Batista da Silva N Jung EM Jung F Schlitt HJ Hornung M. VueBox(R) perfusion analysis of contrast-enhanced ultrasound (CEUS) examinations in patients with primary hyperparathyroidism for preoperative detection of parathyroid gland adenoma. Clin Hemorheol Microcirc. (2018) 70:423–31. doi: 10.3233/CH-189307 30347604

[14] Tranquart F Mercier L Frinking P Gaud E Arditi M. Perfusion quantification in contrast-enhanced ultrasound (CEUS)–ready for research projects and routine clinical use. Ultraschall Med. (2012) 33 Suppl 1:S31–8. doi: 10.1055/s-0032-1312894 22723027

[15] Li N Hu Z Liu Y Ding J Han P Jing X. Dynamic contrast-enhanced ultrasound characteristics of renal tumors: VueBox quantitative analysis. Clin Hemorheol Microcirc. (2023) 85:341–54. doi: 10.3233/CH-231750 37742629

[16] Edmondson HA Steiner PE. Primary carcinoma of the liver: a study of 100 cases among 48,900 necropsies. Cancer. (1954) 7:462–503. doi: 10.1002/1097-0142(195405)7:3<462::aid-cncr2820070308>3.0.co;2-e 13160935

[17] Wang W Wu SS Zhang JC Xian MF Huang H Li W. Preoperative pathological grading of hepatocellular carcinoma using ultrasomics of contrast-enhanced ultrasound. Acad Radiol. (2021) 28:1094–101. doi: 10.1016/j.acra.2020.05.033 32622746

[18] Zhou W Zhang L Wang K Chen S Wang G Liu Z. Malignancy characterization of hepatocellular carcinomas based on texture analysis of contrast-enhanced MR images. J Magn Reson Imaging. (2017) 45:1476–84. doi: 10.1002/jmri.25454 27626270

[19] Claudon M Dietrich CF Choi BI Cosgrove DO Kudo M Nolsoe CP. Guidelines and good clinical practice recommendations for Contrast Enhanced Ultrasound (CEUS) in the liver - update 2012: A WFUMB-EFSUMB initiative in cooperation with representatives of AFSUMB, AIUM, ASUM, FLAUS and ICUS. Ultrasound Med Biol. (2013) 39:187–210. doi: 10.1016/j.ultrasmedbio.2012.09.002 23137926

[20] Rhee H Park YN Choi JY. Advances in understanding hepatocellular carcinoma vasculature: implications for diagnosis, prognostication, and treatment. Korean J Radiol. (2024) 25:887–901. doi: 10.3348/kjr.2024.0307 39344546 PMC11444852

[21] Xu JF Liu HY Shi Y Wei ZH Wu Y. Evaluation of hepatocellular carcinoma by contrast-enhanced sonography: correlation with pathologic differentiation. J Ultrasound Med. (2011) 30:625–33. doi: 10.7863/jum.2011.30.5.625 21527610

[22] Xuan Z Wu N Li C Liu Y. Application of contrast-enhanced ultrasound in the pathological grading and prognosis prediction of hepatocellular carcinoma. Transl Cancer Res. (2021) 10:4106–15. doi: 10.21037/tcr-21-1264 PMC8799228 35116708

[23] Jang HJ Kim TK Burns PN Wilson SR. Enhancement patterns of hepatocellular carcinoma at contrast-enhanced US: comparison with histologic differentiation. Radiology. (2007) 244:898–906. doi: 10.1148/radiol.2443061520 17709836

[24] Salvatore V Gianstefani A Negrini G Allegretti G Galassi M Piscaglia F. Imaging diagnosis of hepatocellular carcinoma: recent advances of contrast-enhanced ultrasonography with sonovue(R). Liver Cancer. (2016) 5:55–66. doi: 10.1159/000367748 29234627 PMC5704684

